# Discovering Cerebral Ischemic Stroke Associated Genes Based on Network Representation Learning

**DOI:** 10.3389/fgene.2021.728333

**Published:** 2021-09-01

**Authors:** Haijie Liu, Liping Hou, Shanhu Xu, He Li, Xiuju Chen, Juan Gao, Ziwen Wang, Bo Han, Xiaoli Liu, Shu Wan

**Affiliations:** ^1^Department of Neurology, Xuanwu Hospital, Capital Medical University, Beijing, China; ^2^Department of Clinical Laboratory, General Hospital of Heilongjiang Province Land Reclamation Bureau, Harbin, China; ^3^Affiliated Zhejiang Hospital, Zhejiang University School of Medicine, Hangzhou, China; ^4^Department of Automation, College of Information Science and Engineering, Tianjin Tianshi College, Tianjin, China; ^5^Department of Neurology, Tianjin Nankai Hospital, Tianjin, China; ^6^Department of Neurology, Baoding No. 1 Central Hospital, Baoding, China; ^7^Graduate School of Chengde Medical College, Chengde, China

**Keywords:** cerebral ischemic stroke, network embedding, disease gene prediction, PPI network, network representation learning

## Abstract

Cerebral ischemic stroke (IS) is a complex disease caused by multiple factors including vascular risk factors, genetic factors, and environment factors, which accentuates the difficulty in discovering corresponding disease-related genes. Identifying the genes associated with IS is critical for understanding the biological mechanism of IS, which would be significantly beneficial to the diagnosis and clinical treatment of cerebral IS. However, existing methods to predict IS-related genes are mainly based on the hypothesis of guilt-by-association (GBA). These methods cannot capture the global structure information of the whole protein–protein interaction (PPI) network. Inspired by the success of network representation learning (NRL) in the field of network analysis, we apply NRL to the discovery of disease-related genes and launch the framework to identify the disease-related genes of cerebral IS. The utilized framework contains three main parts: capturing the topological information of the PPI network with NRL, denoising the gene feature with the participation of a stacked autoencoder (SAE), and optimizing a support vector machine (SVM) classifier to identify IS-related genes. Superior to the existing methods on IS-related gene prediction, our framework presents more accurate results. The case study also shows that the proposed method can identify IS-related genes.

## Introduction

Cerebral ischemic stroke (IS) is the most common type of stroke, which results from a sudden cessation of adequate amounts of cerebral blood supply through vessels (Sacco et al., 2013). As cerebral IS appears to be a complex disorder associated with both genetic and environmental factors, it is highly demanding to demonstrate the underlying patterns of inheritance (Matarin et al., 2010). Some IS-associated genes have been detected, verified, and recorded in recent studies (Cheng et al., 2014). Nevertheless, many unknown cerebral IS-associated genes still need to be discovered. Identifying such genes will significantly contribute to a more detailed understanding of the inherent molecular mechanism of cerebral IS, and will aid the discovery of clinical biomarkers and therapeutic targets. With the development of statistical and machine learning methods in disease-gene discovery, it is crucial to construct and implement a promising computational algorithm for the task of effectively identifying the IS-related genes.

In recent years, predicting disease-related genes has drawn much attention in relative fields and many graph-based computational methods have performed proficiency in integrating large-scale omics data and disease phenotype (Nguyen and Ho, 2012; Zemojtel et al., 2014; Kumar et al., 2018; Wang T. et al., 2020; Peng et al., 2021b). It can be surmised that the prime cost of discovering effective drug targets will be decreased with the engagement of computational algorithms. Under the hypothesis of guilt-by-association (GBA) that most of the existing methods have relied on, it is practicable to explore and even crystallize the unknown disease genes via their connections with the known disease genes (Molet et al., 2013). Based on the GBA hypothesis, disease-associated genes are closely connected or share similar topological structure in the protein–protein interaction (PPI) network. Thus, the effective application of GBA and network-based algorithms largely depends on correct calculation of the distance or similarity between candidate genes and known disease genes.

Many network-based computational methods have also been proposed in recent years (Wang et al., 2019a,b; Yang et al., 2019). For predicting disease genes, one of the initial methods is to simply count the number of disease-genes in the neighborhood of a candidate gene (Oti et al., 2006). However, the direct neighborhood counting methods fail to capture the distant disease genes, i.e., the disease-genes not directly connecting to the candidate gene will be ignored. In this regard, several methods are proposed by considering the distances among genes in a gene network. For instance, methods calculating the shortest path length (SPL) between a candidate gene and the known disease gene have been proposed to examine their biological relatedness. However, Embar et al. (2016) have proved that the average SPL of a gene set only reveals the degree distribution of the genes and their network topology. Thus, methods relying on SPL failed to demonstrate the functional coherence as supposed (Embar et al., 2016). To overcome the shortage of single topological feature in disease-gene prediction, Xu and Li (2006) proposed a method to use multiple topological features together. They integrated five types of local topological features, including degree, 1N index, 2N index, average distance to disease-genes, and positive topology coefficient, and utilized k-nearest neighbors (KNN) as the classifier to distinguish novel disease genes (Xu and Li, 2006). Although the above methods are proven useful, the predicting performance is still not good enough. This is because these methods merely consider local topological features while ignoring the global information. The involvement of global topological information is suggested as a way for obtaining a more impressive gene node presentation and downstream outcomes (Cao et al., 2014; Vuillon and Lesieur, 2015; Peng et al., 2016, 2019).

Considering the global topology information during the learning process is deemed to cause prohibitive computational cost as well as low learning accuracy (Dai et al., 2020). Thus, some studies have tried to develop cost-efficient methods to improve the learning accuracy and explore the multidimensional interactions between genes and proteins with random walk with restart (Valdeolivas et al., 2017; Peng et al., 2019, 2021c). In a recent study, inspired by the idea from random walk with restart, we initiate further application of network representation learning (NRL) that promotes the dimensional reduction of the gene representation in the network and discover the disease-related genes of cerebral IS (Peng et al., 2021a).

In this paper, we utilize the current NRL-based algorithms to predict cerebral IS disease-related genes. Our contributions are three-fold: (1) global topological features of nodes in the PPI network are learned through three cutting-edge graph embedding methods, such as DeepWalk, LINE, and Node2Vec, and their performances are evaluated; (2) the node embeddings are transformed into a low-dimensional space using the deep learning model of a stacked auto-encoder; and (3) we show the superior performance of NRL-based methods for IS gene prediction, and novel genes associated with IS were nominated.

## Methodology

**FIGURE 1:**
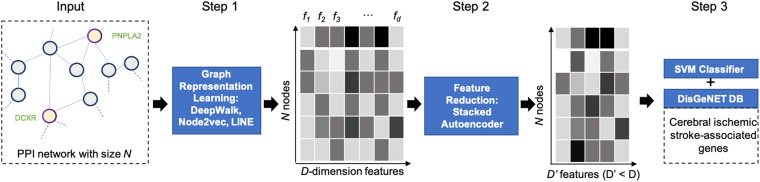
The workflow of the proposed network representation learning (NRL) framework. The framework contains three main parts: Step 1, capturing the topological information of the protein–protein interaction (PPI) network with NRL; Step 2, denoising the gene feature using a stacked autoencoder (SAE); Step 3, training a support vector machine (SVM) classifier to predict IS-related genes.

We apply the NRL-based workflow, as shown in Figure 1, to discover the disease-related genes of IS. The workflow can be concluded into three main parts: extracting features via node representation learning, reducing feature dimension through a stacked autoencoder (SAE; Larochelle et al., 2014), and classification using support vector machine (SVM; Chang and Lin, 2011). First, we utilize three NRL-based algorithms, Node2vec (Grover and Leskovec, 2016), DeepWalk (Perozzi et al., 2014), and LINE (Jian et al., 2015) to collect the high-dimensional feature representation of each gene node from PPI network and compare those structural features captured by different algorithms. In order to avoid the influence of high-dimensional noise, next, we launch a SAE model to map corresponding feature vectors into lower dimensional space. Finally, we use an SVM classifier and convert the process of predicting disease-related genes of IS into node classification problem.

### Graph Embedding for the PPI Network

Based on the need for capturing the global features of topological properties from the PPI network, three classic algorithms (Node2vec, DeepWalk, and LINE) are introduced in the following part. We learn the non-linear feature vectors for genes in the PPI network and compare the performances of the above algorithms.

**FIGURE 2:**
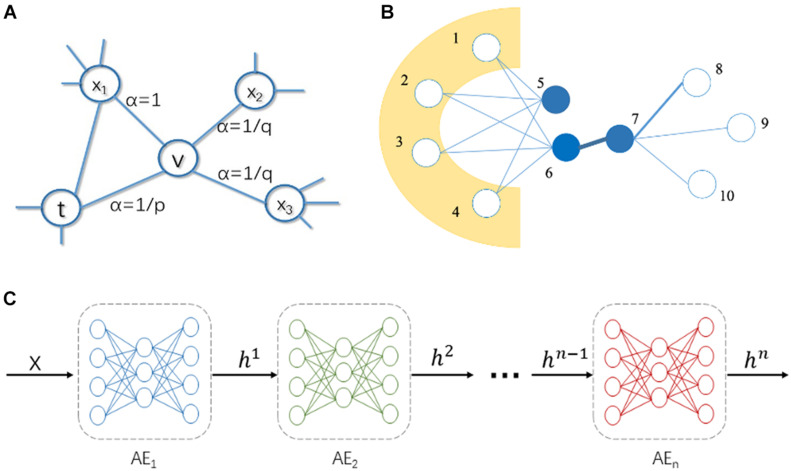
Illustration of Node2vec, LINE, and stacked autoencoder (SAE). **(A)** The biased random walk in Node2vec. **(B)** Illustration of first-order and second order similarity in LINE. **(C)** Structure of SAE, where color represents different layer of the SAE.

DeepWalk serves as the first implemented NRL algorithm and is managed to represent nodes from the PPI network as novel latent feature vectors. At the outset, it runs the classic stochastic process to generate multiple random paths with certain length and this will formulate the topological structure. Then, it can be attributed to a natural language learning process, where the generated random paths are treated as sequences, where nodes are considered as words. Next, the skip-gram neuronal network model is utilized to maximize the probability of neighbors of the nodes in the random walk sequence. In the end, the weight matrix of hidden layer in the skip-gram neuronal network is used as the low-dimensional representation vectors. Node2vec improves DeepWalk algorithm by utilizing a biased random walk process to generate the random paths. It sets hyperparameters *p* and *q* to control the directions of random walk in the manner of breadth-first search (BFS) or depth-first search (DFS), thereby capturing local and global structural features in the network. The function of super parameters *p* and *q* in the random walk procedure is elucidated in Figure 2A. Parameter *p* is called the return parameter, which mainly determines the process of revisiting the nodes within random walk. When *p* is relatively small, the random walk is more inclined to revisit the nodes that have been visited. Parameter *q* is called the in-out parameter, which affects the possibility of capturing “local” or “global” nodes. When *q* > 1, the random walk is inclined to BFS, and when *q* < 1, the random walk is inclined to DFS. Intuitively, the in-out parameter *q* controls the ratio of performing BFS or DFS. Particularly, if *p* and *q* are both equal to 1, the Node2vec algorithm can be simply reckoned as DeepWalk.

Large-scale Information Network Embedding (LINE) is a NRL method based on the assumption of neighborhood similarity, which can be used to learn the low-dimension representation of nodes in a graph. To store network structural information, there are two different definitions of similarity between vertices in a graph. For example, in Figure 2B, there is a strong tie between vertex 6 and 7, so they are two similar vertices. Even if there is no direct correlation between vertex 5 and 6, they share many common neighbors (vertex 1, 2, 3, and 4), which make them the similar nodes.

The two kinds of similarity are described as first-order proximity and second-order proximity. The first-order proximity considers that the greater the edge weight of two vertices, the more similar the two vertices are. Second-order proximity considers that the more common neighbors two vertices have, the more similar the two vertices are.

The first-order proximity in a network is the local pairwise proximity between two vertices. The first-order proximity between u and v is equal to the weight on that edge, *w*_*uv*_. If no edge is observed between u and v, their first-order proximity is 0. For each undirected edge (i,j), the joint probability between vertex *v_i_* and *v_j_* is defined as follows:

(1) $$p_{1}\,\left(v_{i},v_{j}\right)=\frac{1}{1+\text{exp}\,\left(-{\vec{u}}_{i}^{T}\cdot {\vec{u}}_{j}\right)}$$

The empirical probability is defined as ${\hat{p}}_{1}\,\left(i,j\right)=\frac{w_{i\,j}}{W}$, where W = ∑_(*i*,*j*) ∈ *E*_*w*_*ij*_. The objective function is as follows:

(2) $$O_{1}=d\,\left({\hat{p}}_{1}\,\left(\cdot ,\cdot \right),p_{1}\,\left(\cdot ,\cdot \right)\right)$$

The training process is to minimize the KL-divergence of two probability distributions. After replacing d(⋅,⋅) with KL-divergence and omitting some constants, the loss function is:

(3) $$O_{1}=-\sum_{\left(i,j\right)\in E}w_{i\,j}\,\log p_{1}\,\left(v_{i},v_{j}\right)$$

The second-order proximity between a pair of vertices (u,v) in a network is the similarity between their neighborhood network structures. Mathematically, let *p*_*u*_ = (*w*_*u*,1_,…,*w*_*u*,|*V*|_) denote the first-order proximity of u with all the other vertices, then the second-order proximity between u and v is determined by the similarity between *p_u_* and *p_v_*. If no vertex is linked from/to both u and v, the second-order proximity between u and v is 0. For each directed edge (i,j), the probability of “context” *v_j_* generated by vertex *v_i_* can be defined as:

(4) p2⁢(vj|vi)=exp⁢(u→j′T⋅u→i)∑k=1|V|exp⁢(u→k′T⋅u→i)

where |*V*| is the number of vertices or “contexts.”$\vec{u_{i}}$ is the representation of *v_i_* when it is treated as a vertex. ${\vec{u_{i}}}^{\prime }$ is the representation of *v_i_* when it is treated as a specific “context.” The empirical distribution is ${\hat{p}}_{2}\left(\cdot |v_{i}\right)$. So, the objective function is as follows:

(5) $$O_{2}=\sum_{i\in V}\lambda_{i}d\left({\hat{p}}_{2}\left(\cdot |v_{i}\right),p_{2}\left(\cdot |v_{i}\right)\right)$$

λ_*i*_ in the objective function represents the prestige of vertex*i* in the network, which can be measured by the degree or estimated through algorithms such as PageRank. The empirical distribution ${\hat{p}}_{2}\left(\cdot |v_{i}\right)$ is defined as ${\hat{p}}_{2}\left(v_{j}|v_{i}\right)=\frac{w_{i\,j}}{d_{i}}$, where *w*_*ij*_ is the weight of the edge (i,j) and *d_i_* is the out-degree of vertex i, i.e., *d*_*i*_ = ∑_*k* ∈ *N*(*i*)_*w*_*ik*_, where *N*(*i*) is the set of out-neighbors of *v_i_*. After replacing d(⋅,⋅) with KL-divergence, setting λ_*i*_ = *d*_*i*_ and omitting some constants, the loss function is:

(6) $$O_{2}=-\sum_{\left(i,j\right)\in E}w_{i\,j}\,\log p_{2}\,\left(v_{j}|v_{i}\right)$$

The method in this paper is to train the LINE model which preserves the first-order proximity and second-order proximity separately and then concatenate the embeddings trained by the two methods for each vertex.

### Reducing Feature Dimensions Using a Stacked Autoencoder

An autoencoder is an unsupervised model which is well known for its function of extracting features and reducing dimensionality. Aiming at minimizing the reconstruction errors between input and output, an autoencoder consists of two main parts, an encoder and a decoder. The hidden layer encoded features are the final low-dimensional output that plays a vital role in the downstream tasks. If the input node vector is *x*, the reconstructed node vector can be represented as *z*(*x*) = *g*(*w*′⋅*f*(*w*⋅x*b*)*b*′), where *f* and *g* are active functions, *w*,*w*′are weights, and *b*,*b*′ are biases. Hence, the objective function can be represented as Eq. 7, where represents the parameters, and *L* represents the loss function.

(7) $$\theta =\text{arg}\,\min_{\theta }L\,\left(X,Z\right)$$

The SAE is a neural network composed of a multi-layer sparse autoencoder, which is used to boost performance of deep networks, and its structure is shown in Figure 2C. In SAE, the output of the previous layer of autoencoder is used as the input of the next layer of autoencoder. There are three steps to train a SAE. Firstly, a sparse autoencoder is trained on raw input and the trained sparse autoencoder is used to transform the raw input into a feature vector. Secondly, it uses the output of the former layer as input for the subsequent layer and repeats this process until the end of the training. Thirdly, after all the hidden layers are trained, back propagation algorithm is used to minimize the cost function and the pre-trained neural network can be fine-tuned with a labeled training set. SAE has achieved effective outcomes in many areas to extract feature vectors and reduce dimensionality. Alongside this trend, we enroll the SAE model in this proceeding for more impressive performance of predicting IS disease-related genes.

### Predicting Genes Associated With IS Using SVM

After low-dimensional gene features are generated, the SVM algorithm is trained to predict the disease-related genes of IS. The process of predicting such genes is considered as a node classification task. SVM has gained plenty of affirmations for its stability, simplicity, and effectiveness in the way of classification task. Therefore, SVM is engaged in our model analysis. We treat disease-related genes of IS as positive samples, then from the PPI network we randomly designate unlabeled genes of equivalent size as negative samples.

We use five-fold cross validation to evaluate the performance of the SVM classifier in the task of predicting IS disease-related genes. During the experiments, the standard Gaussian kernel is selected for performing the SVM classifier. Besides, we use the grid search method to select the optimal hyper-parameters.

## Results

### Datasets

During the experiments, we downloaded two datasets, the disease-related genes of IS and the PPI network from public resources. The PPI network is originated from Menche et al. (2015), including 13,460 nodes and 141,296 edges. The genes associated with IS were downloaded from the DisGeNet database.^1^ After analyzing and classifying corresponding genes related to IS or cerebral infarction as stated, we finally obtained 1195 IS-related genes.

### Impact of Feature Dimensions on Predicting Performance

**FIGURE 3:**
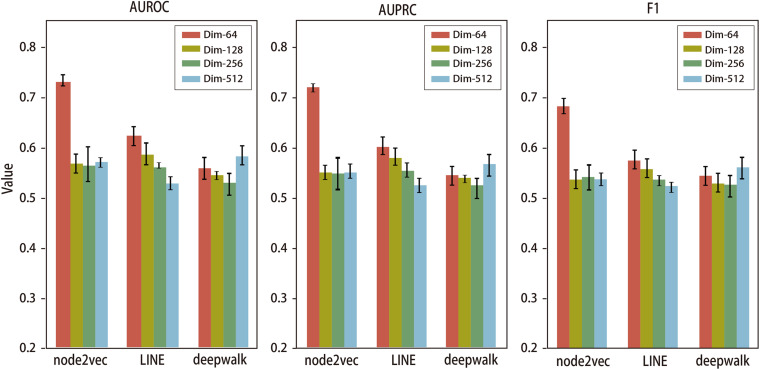
AUROC, AUPRC, and F1 values of node2vec, LINE, and deepwalk using features in different numbers of dimensions. The error bar shows performance variation during the five-fold cross-validation.

In order to explore the optimized dimension of NRL-based algorithms for predicting the disease-related genes of IS, we evaluated the performance of three NRL-based algorithms, i.e., DeepWalk, LINE, and Node2vec, using multiple levels of feature dimensions. Specifically, we run these NRL algorithms to generate features vectors in different dimension-levels, including 64, 128, 256, and 512. All features will be further processed by autoencoder to reduce noise; afterward, the autoencoder will output features in 64 dimensions for downstream predicting tasks. We compared their performance using five-fold cross validation; the results are presented in Figure 3.

We used area under the ROC curve (AUROC), area under the PR curve (AUPRC), and F1 scores to evaluate the performance of deepwalk, LINE, and node2vec in predicting IS-related genes using various feature dimensions. For LINE, the prediction performance drops gradually as the feature dimension increases. For DeepWalk, the prediction performance drops from dim-64 to dim-256, while it increases when feature dimension is up to 512. For node2vec, the best performance is achieved at dim-64 and much better than the other two methods, while other feature dimensions achieve average performance.

**FIGURE 4:**
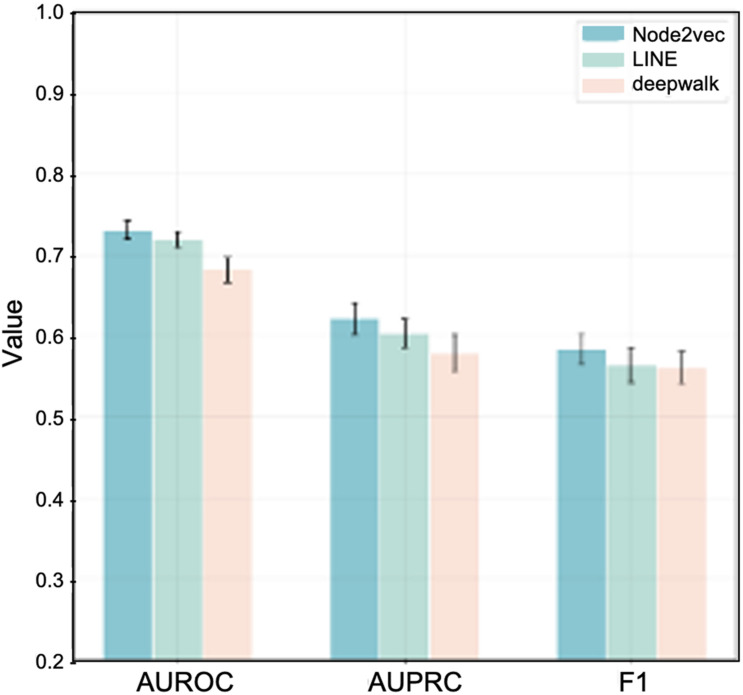
Best performance comparison among three algorithms in the task of IS-related gene prediction.

For intuitional comparison, we summarized the best performance of these three algorithms as shown in Figure 4. We can see that Node2vec with dim-64 provides the most effective outcomes. Therefore, in the final predicting model, we adopt node2vec to learn the graph embedding with 64 feature dimensions.

### Effects of Hyper-Parameters on Ischemic Stroke-Related Gene Prediction

**FIGURE 5:**
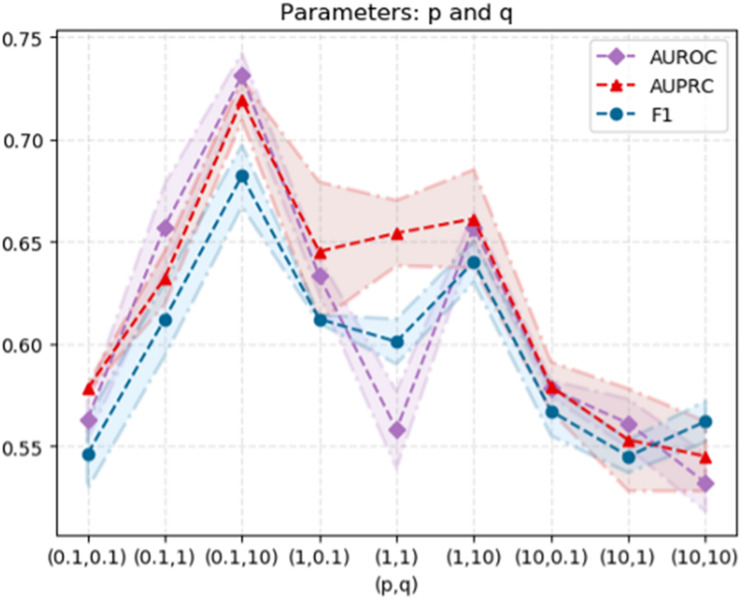
The model performance varies as the hyperparameters (*p*, *q*) change in node2vec.

As mentioned above, the computational workflow use node2vec to capture the topological structure information from the PPI network, followed by extracting low-dimensional features, and predicting disease-related genes based on the SVM classifier. It has been shown in relative researches that the hyper-parameters used in node2vec have considerable impact on the prediction performance. In order to explore the optimized hyper-parameters, we performed a grid search for the hyper-parameters of node2vec, namely *p* and *q*, to test the performance. We randomly select parameters *p* ∈ {0.1, 1, 10} and *q* ∈ {0.1, 1, 10}. When *p* is relatively small, the random walk is more inclined to visit the nodes that have been visited. When *q* > 1, the random walk is biased to BFS, and when *q* < 1, the random walk clings to DFS. The standard deviation of 50% cross validation and the results are shown in Figure 5.

From the data, when *p* = 0.1 and *q* = 10, the AUROC value of the node2vec algorithm achieves its maximum (0.731), which elucidates the optimized choice of hyper-parameters.

### Top Genes Related to Ischemic Stroke

In order to verify the performance of the algorithm in predicting novel genes related to IS, we use existing all-known genes related to IS as the training set and the unknown genes as the test set. Then we rank the probability of final prediction. We select the top 10 genes and list their gene ID and name in Table 1.

**TABLE 1:** Top 10 genes predicted associated with ischemic stroke.

Gene ID	Gene name	Gene description	Score
51181	DCXR	Dicarbonyl and L-xylulose reductase	0.9854
22953	P2RX2	Purinergic receptor P2X 2	0.9762
57104	PNPLA2	Patatin like phospholipase domain containing 2	0.9723
3766	KCNJ10	Potassium inwardly rectifying channel subfamily J member 10	0.9645
3955	LFNG	LFNG O-fucosylpeptide 3-beta-N-acetylglucosaminyltransferase	0.9631
10382	TUBB4A	Tubulin beta 4A class IVa	0.9543
2261	FGFR3	Fibroblast growth factor receptor 3	0.9532
84126	ATRIP	ATR interacting protein	0.9451
2182	ACSL4	Acyl-CoA synthetase long chain family member 4	0.9435
57511	COG6	Component of oligomeric Golgi complex 6	0.9410

Recent studies have shown the correlation between these discovered genes and IS. Cui et al. (2021) utilized lentivirus *in vitro* infection and *in vivo* administration methods to prove that knockdown of ACSL4 alleviated brain injury after IS. Zhao et al. (2020) performed real-time polymerase chain reaction (PCR) to analyze the association between PNPLA2 rs1138693 (T > C) genotype and the risk of IS. Wang J.F. et al. (2020) proved P2RX2 as an up-regulated gene in myocardial infarction using gene ontology (GO) analysis and pathway enrichment analysis in a comparative study of gene expression profiles rooted in acute ischemia and infarction.

### Functional Analysis of the Top Predicted IS-Genes

**FIGURE 6:**
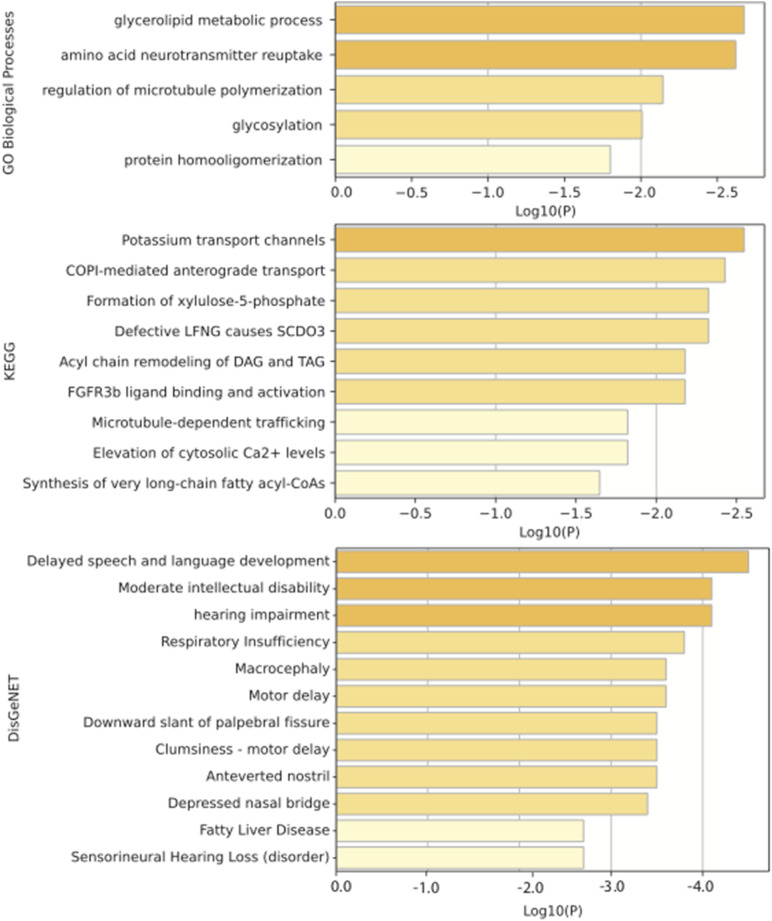
Enrichment analysis of top 10 predicted IS-related genes based on GO, KEGG, and DisGeNet.

We performed enrichment analysis for the top 10 IS-genes predicted by our method based on GO, KEGG, and DisGeNet, and the results are illustrated in the Figure 6. The most GO biological process enriched is the glycerolipid metabolic process. Wang et al. (2021) has proved that the glycerophospholipid metabolism plays a role in IS. KEGG analysis revealed the importance of potassium transport channels in IS, and this also was demonstrated in the work of Chen et al. (2016), where they found that potassium channels can be a potential pharmacological target for IS to slow down cerebral edema formation. The enrichment results from DisGeNet show that the top 10 IS-related genes we predicted are related to language development, intellectual disability, hearing impairment, and motor delays, and these symptoms happen a lot in clinic after occurring IS.

**FIGURE 7:**
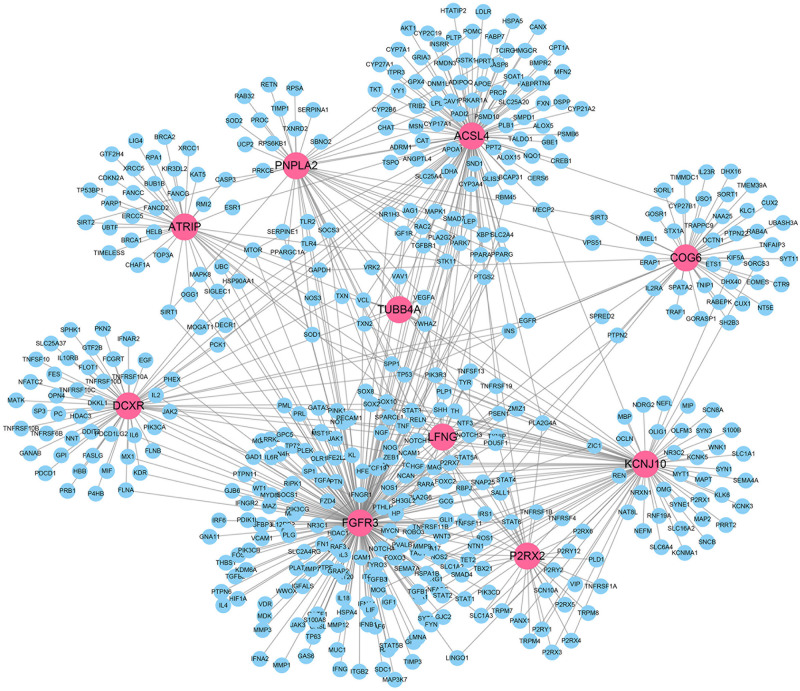
Visualization of gene interactions between predicted IS-genes and existing IS-genes.

We also visualized the gene network between the top 10 predicted IS-genes and the known IS = related genes from DisGeNet in Figure 7. We can see that the top 10 genes predicted by our method are closely connected to the known IS-genes. The gene with highest degree is FGFR3, and the fibroblast growth factors have shown great therapeutic potential in treatment of IS.

## Conclusion

It is quite crucial to discover the disease-related genes of IS for future medical treatment and more accurate diagnosis. In this paper, we utilize NRL methods for the task of identifying disease-related genes and test the novel NRL-based framework to discover IS-related genes. There are three main components in the whole operating process: capturing the global topological information of the PPI, utilizing a SAE to represent vectors into low-dimensional feature space, and training an SVM classifier to predict disease-related genes. The experimental results show that the proposed NRL-based algorithm could achieve considerable accuracy in predicting the genes of IS. Furthermore, the introduced NRL-based algorithms are exploiting and stable to be forwarded to many other fields of potential gene prediction.

## Data Availability Statement

The original contributions presented in the study are included in the article/supplementary material, further inquiries can be directed to the corresponding author.

## Author Contributions

HLiu, LH, and SW conceived the study. HLiu designed and performed the experiments. HLiu, SX, HLi, JG, ZW, and XL analyzed the data and wrote and revised the manuscript. SW supervised the study. All authors contributed to the article and approved the submitted version.

## Conflict of Interest

The authors declare that the research was conducted in the absence of any commercial or financial relationships that could be construed as a potential conflict of interest. The handling editor declared a past co-authorship with one of the authors XL.

## Publisher’s Note

All claims expressed in this article are solely those of the authors and do not necessarily represent those of their affiliated organizations, or those of the publisher, the editors and the reviewers. Any product that may be evaluated in this article, or claim that may be made by its manufacturer, is not guaranteed or endorsed by the publisher.

## Footnotes

1
https://www.disgenet.org/browser/0/1/1/C0026769
